# Bacterial Cyclic Lipopeptides as Triggers of Plant Immunity and Systemic Resistance Against Pathogens

**DOI:** 10.3390/plants14172644

**Published:** 2025-08-25

**Authors:** Ning Ding, Hansong Dong, Marc Ongena

**Affiliations:** 1 Microbial Processes and Interactions (MiPI) Laboratory, TERRA Teaching and Research Centre, Gembloux Agro-Bio Tech, University of Liège, B-5030 Gembloux, Belgium; n.ding@uliege.be; 2 College of Plant Protection, Shandong Agricultural University, Taian 271028, China

**Keywords:** cyclic lipopeptides, induced systemic resistance, plant-beneficial bacteria, biocontrol, plant–microbe interactions, sustainable agriculture

## Abstract

Cyclic lipopeptides (CLPs) are secondary metabolites produced by plant-beneficial bacteria, including *Bacillus*, *Pseudomonas*, *Paenibacillus*, *Burkholderia*, *Serratia*, and *Streptomyces* species. Of these bacterial sources, CLPs from *Bacillus* and *Pseudomonas* have been studied most extensively for their role in plant immunity, particularly in mediating induced systemic resistance. With this review, we provide a unique and comprehensive survey of CLPs from plant-beneficial bacteria described for this function. We consolidate existing knowledge on their role in triggering induced systemic resistance across various plant–pathogen systems and elucidate the underlying mechanisms of symptom suppression. We also discuss the need for further mechanistic studies, but also for implementing each step of the process, leading to marketable CLP-based products used as alternatives to chemicals in sustainable agriculture.

## 1. Introduction

As sessile organisms, plants are particularly vulnerable to environmental threats and have evolutionarily adapted by recruiting plant-beneficial bacteria (PBB) from soil microbiomes. These microbial partners function as biocontrol agents and enhance host resilience via two principal mechanisms, including direct antagonism through the inhibition or elimination of phytopathogens, as well as induction of systemic immune responses that prime the plant for enhanced defense [1,2]. The latter leads to enhanced resistance against subsequent infections, a phenomenon known as induced systemic resistance (ISR). Among PBB, *Pseudomonas* spp. produce a range of small-sized compounds (also referred to as Bioactive Secondary Metabolites, BSMs) that contribute to biocontrol by mediating complex interactions among plants, pathogens, and antagonists. Key BSMs involved in these interactions include phenazines, phloroglucinols, pyoluteorins, pyrrolnitrins, CLPs, hydrogen cyanide, and volatile organic compounds such as nunamycin and nunapeptin [3]. *Bacillus* spp. produce BSMs, including nonribosomally synthesized compounds (e.g., oligopeptides, CLPs, and polyketides) as well as post-translationally modified molecules such as lanthipeptides and bacteriocins. CLPs are particularly interesting and serve as versatile mediators of biocontrol, exhibiting both direct antimicrobial properties and the ability to elicit ISR in plants [4,5,6,7]. Beyond their protective functions, CLPs also play an essential ecological role by supporting bacterial survival and facilitating interactions with other organisms in their environment [8].

In this review, we first briefly characterize CLPs produced by PBB, covering their chemical structures and established biological functions. We then focus specifically on their role as elicitors of ISR in plants. As other metabolites with ISR-inducing activity can be co-produced by bacteria, definitive confirmation of this specific function for CLPs requires the utmost care and strong experimental evidence. This must involve either studies showing loss of function in CLP-deficient mutant strains compared to the wild type and/or bioassays showing systemic resistance induced upon treatment with CLP compounds isolated from bacterial crude extracts, but with sufficient and proven purity (HPLC-purified). Importantly, we exclusively considered studies reporting a clear physical separation between CLPs and pathogens, thereby clearly demonstrating ISR rather than direct antagonism. Common experimental designs include root pretreatment with CLPs or bacterial strains followed by leaf pathogen challenge (using either seedlings or detached leaves), local leaf treatment with CLPs/bacteria followed by distal leaf pathogen inoculation, or soil-based root treatment with subsequent stem pathogen application. For studies deviating from these approaches, demonstration of negative direct antagonism (as in references [9,10]) is required to confirm ISR activity. We also exclusively consider studies that investigate CLP activity at physiologically relevant concentrations (a low micromolar range), thereby reflecting natural biological conditions.

This review provides a comprehensive analysis of CLP-induced ISR through multiple perspectives: structural characteristics and effective concentrations of CLPs; susceptible plant and pathogen species; and current understanding of perception mechanisms. We specifically address CLP-mediated ISR across diverse plant systems. We highlight the untapped potential of these compounds and advocate for intensified research efforts to fully characterize CLP properties for sustainable agricultural applications.

## 2. CLP Biosynthesis

**Figure 1:**
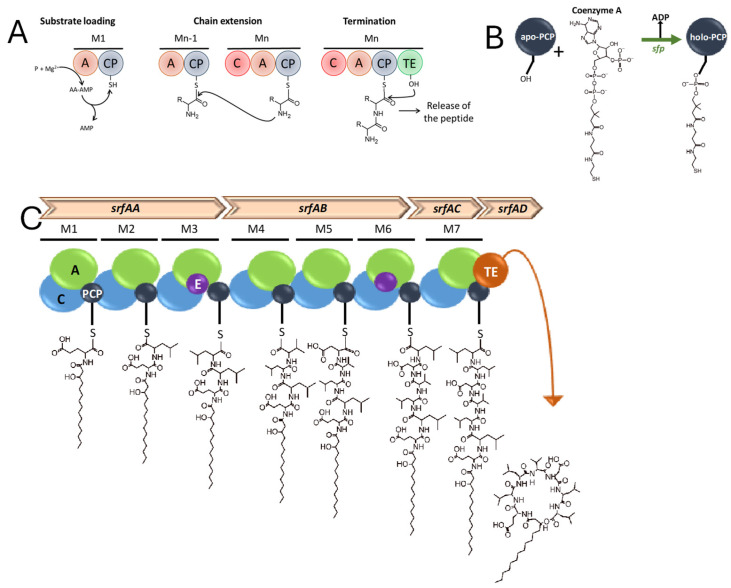
Modular organization and main steps in the nonribosomal biosynthesis of surfactin as a model CLP. (**A**) Modules and main steps in biosynthesis of nonribosomal peptides. Adenylation (A), peptidyl carrier protein (CP), and condensation (C) domains act as an assembly line. The thioesterase (TE) domain of the termination module cleaves the thioester bond and releases the peptide. (**B**) Activation of the CP domain of the NRPS, where the serine residue of unactivated PCP (apo-PCP) has to be combined with coenzyme A, thanks to the 4′phosphopanteteinyl transferase, encoded by the *sfp* gene, to be able to play its carrier role (holo-PCP). (**C**) Representation of the whole surfactin assembly line.

CLPs are produced through nonribosomal peptide synthetases (NRPSs). These multi-modular enzyme complexes are encoded by well-characterized giant biosynthetic gene clusters (BGCs) [11,12], which have been identified and characterized in *Bacillus* and *Pseudomonas* species [13]. NRPSs function as sophisticated molecular assembly lines, incorporating amino acid (AA) residues and facilitating various cyclization reactions [12]. The NRPS architecture typically comprises several functional domains: adenylation (A) activates AA substrates; the thiolation/peptidyl carrier protein (T/PCP) transports growing peptide chains; condensation (C) catalyzes peptide bond formation; starter condensation (Cs) mediates lipid–peptide conjugation at the N-terminus; epimerization (E) converts L- to D-AAs; and thioesterase (TE) terminates elongation and facilitates cyclization [14,15,16] (Figure 1). The CLP biosynthetic pathway involves three key stages: precursor activation, sequential peptide chain elongation with concurrent FA incorporation, and final export of the mature metabolite [17]. For most strains, CLPs are biosynthesized exclusively through NRPS-mediated thiotemplate mechanisms [18]. However, *Bacillus* species employ distinct pathways for different CLP families: the Surfactin and Fengycin families, containing β-hydroxy FAs, are produced by dedicated NRPS systems; the iturin family, characterized by β-amino FAs, is synthesized via a polyketide synthase (PKS)–NRPS hybrid pathway [19]. The locillomycin biosynthetic machinery also employs a PKS-NRPS hybrid complex that substitutes the canonical Cs domain with an integrated PKS acyl ligase (AL) domain for FA incorporation [20].

## 3. Diversity of CLP Structures and Functions

**Figure 2:**
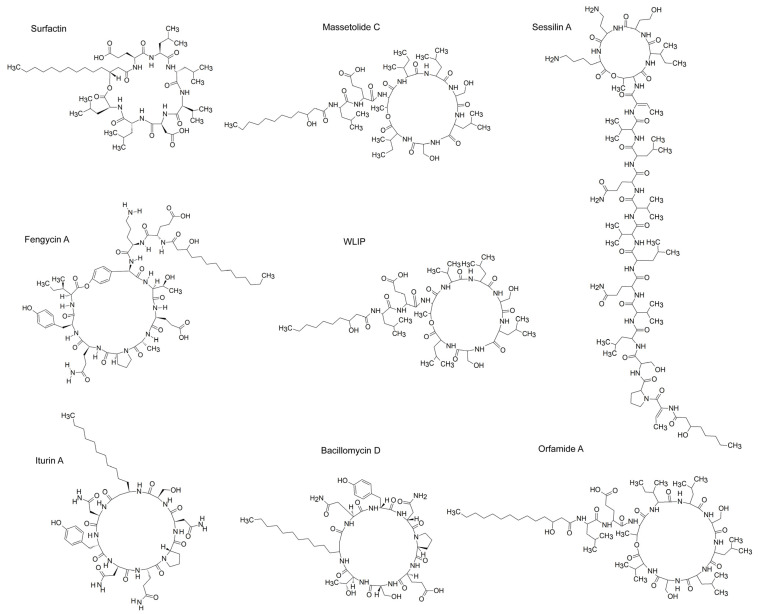
Chemical structures of main CLPs with ISR-inducing activity. The figure represents the mentioned CLPs (surfactin, fengycin A, iturin A, massetolide C, WLIP, bacillomycin D, sessilin A, and orfamide A) drawn with ChemSketch 12.01.

PBBs represent a phylogenetically diverse group of rhizosphere-associated microorganisms that promote crop productivity. Major PBB genera include *Bacillus*, *Pseudomonas*, *Acetobacter*, *Azospirillum*, *Paenibacillus*, *Serratia*, *Burkholderia*, *Herbaspirillum*, *Rhodococcus*, *Actinobacteria*, and *Lactobacillus* [21]. Many PBBs can produce CLPs, but this review primarily focuses on CLPs from *Bacillus* and *Pseudomonas* spp., which are by far the best described (Table 1 and Figure 2).

### 3.1. Bacillus CLPs

*Bacillus*-derived CLPs are classified into three main families: Surfactins (including surfactin, pumilacidin, lichenysin, bamilocyn, and halobacilin), Fengycins (fengycin/plipastatin and maltacin), and Iturins (iturin, mycosubtilin, bacillomycin, mojavensin, mixirins, and bacillopeptins) [22,23,24]. Additional structurally distinct CLPs include kurstakin, locillomycin, antiadhesin, circulocin, and licheniformin [24]. These compounds exhibit variations in both their FA moieties (ranging from β-hydroxy to β-amino forms, with either ester or amide linkages) and their cyclic peptide structures (comprising 5–10 AA residues in various L/D configurations) [24]. The β-hydroxy-type CLPs, including surfactin, fengycin, kurstakin, antiadhesin, bamylocin A, circulocin, and locillomycin, predominantly feature ester bonds, while β-amino-type compounds like iturin and licheniformin contain characteristic amide linkages. Notably, some CLPs such as circulocins incorporate unusual guanylated FA chains [24]. Functionally, each CLP family displays distinct antimicrobial profiles. Surfactins exhibit some limited activity against both bacteria and fungi, while iturins and fengycins demonstrate particularly potent antifungal properties [23,25].

### 3.2. Pseudomonas CLPs

CLPs produced by *Pseudomonas* species can be categorized based on whether they are derived from beneficial strains (plant growth-promoting rhizobacteria or biocontrol agents) or pathogenic strains (phytopathogens). This division highlights the dual roles of *Pseudomonas* CLPs, either as biocontrol tools in sustainable agriculture or as virulence determinants in plant diseases, depending on the ecological nature of the producing strain [26].

Beneficial *Pseudomonas* strains, such as *P. fluorescens*, *P. putida*, and *P. protegens*, produce CLPs that contribute to biocontrol, biofilm formation, and plant growth promotion [27]. These include the viscosin group (e.g., viscosin, viscosinamide, massetolide, WLIP, pseudodesmin, and pseudophomin), the amphisin group (e.g., lokisin/anikasin, milkisin/stechlisin/tensin, arthrofactin, pholipeptin, amphisin, oakridgin, and nepenthesin), and the bananamide group (e.g., bananamide A-G, MDN-0066, and prosekin) [27]. Additional CLPs from beneficial strains include orfamide, putisolvin, gacamide, xantholysin, entolysin, and cocoyamide, which exhibit antimicrobial and surfactant properties that support their role in microbial competition and root colonization [26,27,28].

In contrast, pathogenic *Pseudomonas* strains, such as *P. syringae*, *P. tolaasii*, and *P. corrugata*, produce CLPs that function as virulence factors that promote host cell lysis through pore formation, disrupting host cell membranes and promoting disease [28]. Pathogenic *Pseudomonas* CLPs primarily belong to the Mycin (e.g., syringotoxin) and Peptin (e.g., fuscopeptin) families [28]. The syringomycin group (e.g., syringomycin, syringostatin, pseudomycin A, cormycin A, syringafactin, cichofactin, virginiafactin, and thanamycin) is particularly notable for its phytotoxic effects [27]. Similarly, the tolaasin group (e.g., tolaasin, sessilin, corpeptin, and FP-B) induces necrosis in plants, and the poaeamide group (e.g., PPZPM and poaeamide A) is associated with pathogenic *P. poae* [27].

### 3.3. CLPs from Other Bacterial Genera

Besides *Bacillus* and *Pseudomonas* species, numerous soil-dwelling bacteria that contribute to plant health and ecological adaptation also produce CLPs. Among these, *Paenibacillus*-derived CLPs exhibit remarkable structural and functional diversity. Fusaricidin, a non-cationic lipopeptide, features a hexapeptide ring with both ester and amide linkages, along with a guanidinylated β-hydroxy FA moiety [29,30]. Structural variations among fusaricidins arise from AA substitutions at three positions within the peptide ring, conferring activity against fungi and Gram-positive bacteria [30]. *Paenibacillus* also produces cationic CLPs, including the polymyxin family (polymyxin/colistin A, B, C, D, E; mattacin/M; P, S, T), which vary in AA composition at specific residue positions; octapeptins (e.g., battacin) that exhibit broad-spectrum antimicrobial activity with reduced toxicity; the 13-AA-residue paenibacterin that disrupts both outer and cytoplasmic membranes; and other structurally distinct cationic CLPs such as polypeptins (e.g., pelgipeptin), paenibacterin, and gavaserin [30,31,32]. Notably, octapeptins demonstrate efficacy against both Gram-negative and Gram-positive bacteria through mechanisms distinct from classical polymyxins [30,31,32].

*Serratia* species synthesize structurally distinct CLPs, primarily from the Serrawettin family (serratamolide A to G; serrawettin W2 to W3). Serratamolide A (serrawettin W1) features a symmetric dilactone structure with two β-hydroxy FAs linked to two AAs (L-Ser_1_-L-Ser_2_), displaying antitumor, antimicrobial, and plant-protective properties [33,34,35]. Structural variations among serrawettin homologs (serratamolide B–G) arise from FA chain lengths (C_8_-C_14_) and unsaturation patterns [33]. In contrast, serrawettin W2 contains a pentapeptide core (D-Leu_1_-L-Ser_2_-L-Thr_3_-D-Phe_4_-L-Ile_5_) [33]. The stephensiolide family (A–K), originally identified from mosquito-associated *Serratia* sp., combines antibiotic activity with roles in bacterial motility. Genomic analyses suggest that these compounds may facilitate ecological niche colonization [36].

*Actinomycetia*-class bacteria represent a significant source of structurally diverse CLPs with notable bioactivities. *Kitasatospora cystarginea* produces cystargamides, characterized by a hexapeptide ring linked to an unusual 2,3-epoxy FA (C_10_) chain. Notably, cystargamide B has emerged as a promising lead compound for dengue fever treatment [37]. *Actinoplanes* species synthesize ramoplanin, a lipoglycodepsipeptide antibiotic featuring a 16-AA cyclic peptide conjugated to C_8_-C_10_ FA, demonstrating potent activity against Gram-positive bacteria [38]. *Streptomyces* species exhibit remarkable biosynthetic capacity and produce numerous CLPs including the clinically approved antibiotic daptomycin (Cubicin^®^) containing a 13-AA cyclic structure, some calcium-dependent antibiotics (CDAs; A54145), and other structurally distinct compounds (amphomycin, laspartomycin, and arylomycins) [38,39,40].

## 4. CLPs as Inducers of Plant-Species-Dependent ISR

*Bacillus*-derived CLPs from the Surfactin, Fengycin, and Iturin families demonstrate varying capacities in activating ISR depending on both the host plant and challenging pathogen. Among them, surfactin is the most well studied for triggering ISR in many plants, especially dicotyledons, such as in bean, tomato, grapevine, *Arabidopsis*, and tobacco against *Botrytis cinerea*; in melon against *Podosphaera fusca*; in strawberry against *Colletotrichum gloeosporioides*; in peanut against *Sclerotium rolfsii* and *Athelia rolfsii*; and in *Arabidopsis* against *Pseudomonas syringae* [9,41,42,43,44,45,46,47,48,49,50,51]. Notably, comparative studies reveal functional specialization among CLP families. In melon plants, fengycin and iturin mutants fail to induce ISR against *Podosphaera fusca*, unlike surfactin [42]. A parallel response is observed in grapevine plants, where plipastatin (a Fengycin-family CLP) does not trigger ISR against *Botrytis cinerea*, contrasting with surfactin’s activity [9].

Fengycin has also been demonstrated to induce ISR in multiple plant systems, such as in tomato against *Botrytis cinerea*, in *Arabidopsis* against *Pseudomonas syringae*, and in Chinese cabbage against *Plasmodiophora brassicae* [41,46,52,53]. Similarly, the Iturin-family CLPs (iturin and bacillomycin) exhibit ISR-eliciting activity across diverse hosts: iturin mediates protection in strawberry against *Colletotrichum gloeosporioides*, chili pepper against *Phytophthora capsici*, and *Arabidopsis* against *Pseudomonas syringae* [44,50,54], while bacillomycin induces ISR in *Arabidopsis* against both *Pseudomonas syringae* and *Botrytis cinerea* [46]. Intriguingly, in the rice–*Pyricularia oryzae* pathosystem, synergistic application of fengycin and iturin (with or without surfactin) is required for ISR induction, independent of soil conditions (acid sulfate or healthy potting soil) [55].

The induction of systemic resistance by CLPs exhibits some host specificity. While 5 µM surfactin effectively triggers ISR in bean plants against *Botrytis cinerea* and in peanut plants against *Sclerotium rolfsii* [41,47], this concentration fails to induce resistance in tomato plants against *Botrytis cinerea* [49]. Notably, fengycin demonstrates complementary activity patterns, showing no ISR induction in bean plants regardless of application method (using either fengycin-overproducing strains or a 5 µM purified compound), but effectively priming resistance in tomato plants when applied via fengycin-deficient bacterial strains [41]. Consistent with the existing literature, data reveal surfactin’s unique role as the only tested lipopeptide capable of eliciting ISR against *Podosphaera fusca* in melon, distinguishing it from fengycin and iturin [42]. Together, these results highlight the complex interplay between combinations of CLP structure and the host plant’s pathogen in determining ISR efficacy.

Current research demonstrates that *Pseudomonas*-synthesized CLPs can elicit ISR in a highly pathosystem-dependent manner. Massetolide A primes tomato defenses against *Phytophthora infestans* infection [56], while sessilin and orfamide, either individually or in combination, induce protective responses in bean plants challenged with *Rhizoctonia solani* [57]. Striking specificity is observed in rice systems, where orfamide confers resistance to *Cochliobolus miyabeanus* but not *Magnaporthe oryzae*, and the structurally distinct CLP WLIP shows opposing selectivity against *Magnaporthe oryzae* [58,59]. These findings collectively underscore that CLP-mediated ISR activation depends critically on precise molecular recognition events between specific CLP structures and particular plant–pathogen combinations. Table 2 shows CLPs from *Bacillus* and *Pseudomonas* species eliciting ISR across diverse plant–pathogen systems.

## 5. Differential Activation of ISR by CLPs Across Concentration Gradients

The capacity of CLPs to elicit ISR exhibits striking concentration dependence and structural specificity. In *Arabidopsis thaliana*, surfactin demonstrates pathogen-specific activation thresholds that 1 µM suffices for protection against *Botrytis cinerea* but fails against *Pseudomonas syringae*, while intermediate concentrations (2–16 µM) are effective against the bacterial pathogen *Pseudomonas syringae*, but higher concentrations at 32 µM are not [45,50]. Most studies report optimal ISR induction at 10 µM for surfactin across diverse pathosystems, including in melon plants against *Podosphaera fusca*, in *Arabidopsis*, tobacco, grapevine plants against *B. cinerea*, and in peanut plants against *Sclerotium rolfsii* and *Athelia rolfsii* [9,42,45,47,48,51]. There is thus a clear concentration–response relationship. While 5 µM surfactin is ineffective in tomato plants, 25 µM provides protection against *Botrytis cinerea* [49]. Similar dose–responses occur with other CLPs like orfamide, requiring more than 25 µM for ISR in rice plants against *Cochliobolus miyabeanus* [58]. Iturin shows a narrow effective window (0.5–2 µM) in *Arabidopsis* against *Pseudomonas syringae*, unlike at a lower concentration of 0.25 µM and a higher concentration of 4 µM [50]. Higher iturin concentrations (10 µM) are needed to trigger ISR in strawberry infected with *Colletotrichum gloeosporioides* [44]. These findings highlight our limited understanding of how CLP structural features govern ISR activation. The concentration-dependent effects and pathogen-specific induction patterns imply the existence of a sophisticated perception mechanism that requires further molecular characterization.

## 6. Synergistic Relationship of CLP Application Triggering ISR in Plants

Growing evidence indicates that different CLPs can act synergistically to enhance their biological functions. While single CLP-mediated ISR has been well-documented, recent studies highlight the effect of CLP combinations in modulating plant immune responses. For instance, iturin and surfactin cooperate to promote robust biofilm formation [60], while mixtures of fengycin and surfactin induce stronger ISR in bean plants against *B. cinerea* than either CLP alone [41]. Similarly, when combined, sessilin and orfamide exhibit synergistic activity against *Rhizoctonia solani* in bean plants [57].

ISR induction in rice plants against *Pyricularia oryzae* strictly requires the combined application of fengycin and iturin, irrespective of soil conditions [55]. Notably, surfactin that fails to trigger ISR alone potentiates the effect of fengycin–iturin mixtures, revealing complex higher-order interactions among CLPs [55]. These observations challenge the traditional single-component view of CLP activity and instead suggest that natural CLP mixtures may function cooperatively to elicit immune responses beyond what individual compounds can achieve.

Such synergistic effects underscore the ecological relevance of CLP diversity in rhizosphere communities and highlight the need to reconsider biocontrol strategies using rationally designed CLP combinations. However, the molecular mechanisms underlying these interactions remain poorly understood, emphasizing a key gap in our knowledge of how these microbial metabolites collectively shape plant immunity.

## 7. Molecular Mechanisms of CLP Recognition in Plants

Plants detect microbial presence through plasma membrane-localized pattern recognition receptors (PRRs) that perceive conserved microbial-associated molecular patterns (MAMPs), initiating pattern-triggered immunity (PTI) [61]. In contrast to this direct defense response, PBBs typically induce a more subtle priming ISR to enhance readiness for future pathogen challenges without immediate metabolic costs. While PTI triggers rapid, energy-intensive defense responses, ISR-mediated priming provides targeted protection that activates fully only upon subsequent pathogen recognition. This distinction is exemplified by surfactin’s mode of action in *Arabidopsis thaliana*. Unlike classical MAMPs (e.g., flagellin), surfactin neither elicits reactive oxygen species (ROS) bursts nor strongly upregulates defense-related genes during initial perception [45]. Instead, it establishes a primed state that enables amplified defense responses upon actual pathogen encounter.

The biological activity of CLPs stems from their specific interactions with cell membrane components. As amphiphilic molecules, CLPs spontaneously integrate into plasma membranes, whose complex structures are composed of phospholipids, sphingolipids, and sterols, through thermodynamically favorable hydrophobic interactions [62,63]. These interactions exhibit remarkable structural specificity that governs CLP bioactivities and their immune-modulating effects. Mycosubtilin demonstrates selective affinity for ergosterol in fungal membranes [64], while surfactin is preferentially incorporated into phospholipid bilayers, inducing subtle membrane perturbations rather than outright disruption. This delicate structural modulation appears sufficient to initiate downstream signaling cascades culminating in ISR [4,41,48,65,66]. Fengycins, by contrast, exert their effects through more generalized alterations of membrane architecture and permeability [41,65]. Structure–activity studies reveal critical determinants of CLP functionality. The bioactivity of iturin derivatives depends on both the integrity of their cyclic peptide structure and their FA chain composition, as demonstrated in *Arabidopsis thaliana* [67]. Similarly, among surfactin homologs, those with longer FA chains (C_14_-C_15_) show superior immune-stimulating capacity in tobacco cells compared to shorter chain variants (C_12_-C_13_) or structurally modified derivatives (linearized or methylated peptides) [68]. These in vitro observations highlight the exquisite structure–function relationships governing CLP activity, though their physiological relevance requires further validation in plants.

The biological activity of CLPs is fundamentally governed by their unique structural architecture. Cyclization of the peptide backbone confers enhanced molecular stability [69] and is essential for optimal bioactivity, as demonstrated by the consistently reduced efficacy of linear analogs. This structural requirement reflects the critical importance of three-dimensional conformation for target engagement, where the precise spatial arrangement of functional groups determines interaction specificity. CLP–membrane interactions exhibit remarkable structural selectivity based on several key physicochemical parameters. The amphipathic character of these molecules, dictated by FA chain length (typically C_12_-C_15_), branching patterns, and peptide ring composition (including AA stereochemistry), collectively determines their membrane insertion dynamics and subsequent biological effects [62]. These structure–activity relationships explain why subtle modifications, such as methylation or linearization of the peptide chain, can significantly alter immune-stimulating capacity [68]. However, further investigation is required to better understand the impact of the chemical structure of CLPs on their ISR functionality. For instance, additional structural analogs with variations in the peptide sequence should be tested. Interestingly, fluorinated CPL analogs display enhanced antimicrobial activity, and such derivatives could be tested for ISR [70,71].

Recent mechanistic studies have revealed unconventional perception pathways for certain CLPs. Surfactin’s dose-dependent activity in the micromolar range, coupled with its ability to modulate membrane fluidity without receptor binding, suggests a lipid-driven signaling mechanism [68]. This is further supported by its newly identified role in regulating miR846-mediated jasmonic acid signaling [72], representing a novel interface between membrane biophysics and transcriptional reprogramming. However, significant knowledge gaps remain in our understanding of CLP-mediated immunity. The molecular sensors responsible for CLP detection in plants have not been fully characterized, and the downstream signaling networks connecting membrane perturbations to systemic responses remain poorly mapped [41,65]. Furthermore, the translation of in vitro structure–activity relationships to whole-plant systems requires rigorous validation, particularly regarding concentration thresholds and tissue-specific effects.

## 8. Conclusions

CLPs demonstrate remarkable environmental stability, maintaining their structural and functional integrity under extreme temperature, salinity, and pH conditions [17]. While their role as plant immunity elicitors has been established, the current research landscape reveals significant geographical and taxonomic biases. Studies on *Bacillus*-derived CLPs dominate the literature, with 18 publications demonstrating their ISR-inducing capacity, primarily from research groups in Belgium (6 papers) and China (4 papers), followed by contributions from Japan, France, Spain, South Korea, Argentina, and Chile. In contrast, investigations into *Pseudomonas* CLPs remain limited to a single Belgian research group, with publication peaks occurring triennially between 2015 and 2021. The absence of new studies since 2024 highlights an urgent need for broader international engagement in this field.

Substantial gaps persist in our understanding of CLP-mediated ISR. Despite established ISR effects of surfactin/fengycin in wheat *(Zymoseptoria tritici*) [10], fengycin/iturin in wheat (*Fusarium graminearum*) [73], and fengycin in tomato (*Sclerotinia sclerotiorum* pathosystem) [74], further studies are still needed to evaluate CLP efficacy at lower, physiologically relevant concentrations. Furthermore, the current research focus remains narrowly confined to *Bacillus* and *Pseudomonas* CLPs, despite the phylogenetic diversity of rhizobacteria capable of CLP production. Expanding investigations to understudied bacterial taxa could reveal novel CLP structures with unique bioactivities.

The dual roles of CLPs as both phytotoxins and immune elicitors are exemplified by contrasting systems. Pathogenic *Pseudomonas cichorii* SF1-54 produces cichopeptins (Peptin-family CLPs) that contribute to lettuce midrib rot virulence [75], whereas *Pseudomonas brassicacearum* R401′s brassicapeptin A induces stress symptoms in salt-treated plants despite its non-pathogenic origin [76]. This paradox highlights the context-dependent nature of CLP–plant interactions. This indicates the complexity of plant–microbe interactions, where not all pathogen-derived molecules are purely virulent, as some can stimulate host defenses in specific contexts; dose and environment critically determine whether a CLP functions as a virulence factor or an immune elicitor. Additional studies are required to better understand the links between structure, dose, and the impact of the various CLPs on plant cells in terms of membrane damage and immune activation responses.

Genetic evidence demonstrates distinct jasmonic acid-dependent pathways for CLP-mediated immunity in *Arabidopsis*. Surfactin induces ISR against *Pseudomonas syringae* specifically in *jar1-1* mutants but not wild-type or *npr1* plants. Iturin shows broader efficacy in both wild-type and *jar1-1* backgrounds while remaining inactive in *npr1* mutants [50]. These findings reveal crucial dependencies on both JAR1-mediated jasmonic acid signaling and NPR1-dependent salicylic acid pathways for different CLP classes. While this review synthesizes current understanding of CLP perception and ISR induction, critical knowledge gaps remain regarding structure–function relationships. Genome-wide transcriptomics and metabolomics would help elucidate the molecular determinants governing these differential responses, particularly how specific CLP structures engage distinct signaling modules. This could be essential for developing targeted crop protection strategies. Previously unresolved questions highlight the need for innovative approaches combining structural biology, membrane biophysics, and plant immunology. This will refine our understanding of the process mediating CLP perception by plant cells via membrane interactions and elucidate the signal transduction cascades activated by different CLP structural classes.

CLPs retain a strong potential for application in plant protection, but no product based on these compounds has been commercialized so far. There are several reasons for this, but relatively low productivity by the bacteria, coupled with high production costs in bioprocesses, certainly constrain such commercial development of low added-value products for agricultural applications [77,78]. Some advances have been made in CLP production and purification [77,78], but further optimization of each step of cost-effective processes at an industrial scale is mandatory for scalable manufacturing and widespread use of these “green compounds”. It first includes enhancing cell productivity in bioreactors via precursor feeding, metabolic engineering, and/or culture media and fermentation condition optimization [79,80,81,82]. CLP recovery and purification from large fermentation volumes, as well as their formulation, usually represent a significant part of total production cost and must also be optimized, taking advantage of membrane- and chromatography-based technologies relying on recent advances [83]. Next, formulated CLP-based products must be further evaluated for their ecotoxicity and for their efficacy under field conditions when applied as a single product or in an IPM (integrated pest management) approach. Indeed, nearly all studies have been conducted under controlled healthy soil conditions, with only one exception [55] considering the complex interplay between CLPs and environmental stressors. Future research must incorporate biotic and abiotic variables to better predict field performance. These advances will bridge the gap between mechanistic understanding and practical implementation of CLP and will significantly enhance our ability to harness these molecules for sustainable crop protection strategies.

## Figures and Tables

**Table 1:** Simplified primary chemical structures of CLPs reported to trigger ISR in plants. *a*Thr refers to *allo*-threonine. The underlined AA residues refer to the AAs in the cyclic peptide ring. The red color marks D-AAs, and the rest are L-AAs. So far, the determination of the AAs of sessilin A to be L or D form is not clear.

CLPs	FA	AA																	
		1	2	3	4	5	6	7	8	9	10	11	12	13	14	15	16	17	18
Surfactins	C_12_-C_17_	Glu	Leu	Leu	Val	Asp	Leu	Leu	/	/	/	/	/	/	/	/	/	/	/
Fengycins	C_14_-C_18_	Glu	Orn	Tyr	* a * Thr	Glu	Ala	Pro	Gln	Tyr	Ile	/	/	/	/	/	/	/	/
Iturins	C_14_-C_17_	Asn	Tyr	Asn	Gln	Pro	Asn	Ser	/	/	/								
Bacillomycins	C_14_-C_17_	Asn	Tyr	Asn	Pro	Glu	Ser	Thr	/	/	/								
Massetolide A	C_10_	Leu	Glu	*a*Thr	*a*Ile	Leu	Ser	Leu	Ser	Ile	/	/	/	/	/	/	/	/	/
WLIP	C_10_	Leu	Glu	*a*Thr	Val	Leu	Ser	Leu	Ser	Ile	/	/	/	/	/	/	/	/	/
Sessilin A	C_8_	Dhb	Pro	Ser	Leu	Val	Gln	Leu	Val	Val	Gln	Leu	Val	Dhb	Thr	Ile	Hse	Dab	Lys
Orfamide A	C_14_	Leu	Glu	*a*Thr	Ile	Leu	Ser	Leu	Leu	Ser	Val	/	/	/	/	/	/	/	/

**Table 2:** CLPs reported to trigger ISR in plants.

CLP Name	Strain Species	Plant	Pathogen	Method	Reference
Surfactin	*Bacillus subtilis*	Bean	*Botrytis cinerea*	Pure compound; mutants	[41]
Surfactin	*Bacillus subtilis*	Tomato	*Botrytis cinerea*	Mutants	[41]
Surfactin	*Bacillus subtilis*	Melon	*Podosphaera fusca*	Mutant; mutant and commercial C_15_ surfactin; commercial C_15_ surfactin	[42]
Surfactin	*Bacillus velezensis; Bacillus subtilis; Paenibacillus polymyxa*	Tomato	*Botrytis cinerea*	Strains producing different amounts of surfactin	[43]
Surfactin	/	Strawberry	*Colletotrichum gloeosporioides*	Pure compound	[44]
Surfactin	*Bacillus subtilis*	Grapevine	*Botrytis cinerea*	Pure compound	[9]
Surfactin	*Bacillus velezensis*	*Arabidopsis*	*Botrytis cinerea*	Pure compound	[45]
Surfactin	*Bacillus amyloliquefaciens SQR9*	*Arabidopsis*	*Botrytis cinerea*	Mutant	[46]
Surfactin	*Bacillus amyloliquefaciens SQR9*	*Arabidopsis*	*Pseudomonas syringae*	Mutant	[46]
Surfactin	*Bacillus subtilis*	Peanut	*Sclerotium rolfsii*	Pure compound	[47]
Surfactin	*Bacillus velezensis*	Tobacco	*Botrytis cinerea*	Pure compound	[48]
Surfactin	/	Tomato	*Botrytis cinerea*	Commercial compound	[49]
Surfactin	/	*Arabidopsis*	*Pseudomonas syringae*	Commercial compound	[50]
Surfactin	/	*Arabidopsis jar1-1*	*Pseudomonas syringae*	Commercial compound	[50]
Surfactin	*Bacillus subtilis*	Peanut	*Athelia rolfsii*	Pure compound	[51]
Fengycin	*Bacillus subtilis*	Tomato	*Botrytis cinerea*	Mutant	[41]
Fengycin	*Bacillus velezensis*	*Arabidopsis*	*Pseudomonas syringae*	Mutant	[46]
Fengycin H	*Bacillus cabrialesii*	Tomato	*Botrytis cinerea*	Pure compound	[52]
Fengycin	*Bacillus subtilis*	Chinese cabbage	*Plasmodiophora brassicae*	Mutant	[53]
Iturin A	/	Strawberry	*Colletotrichum gloeosporioides*	Commercial compound	[44]
Iturin A	*Bacillus vallismortis*	Chili pepper	*Phytophthora capsici*	Pure compound	[54]
Bacillomycin D	*Bacillus velezensis*	*Arabidopsis*	*Pseudomonas syringae*	Mutant	[46]
Bacillomycin D	*Bacillus velezensis*	*Arabidopsis*	*Botrytis cinerea*	Mutant	[46]
Iturin A	*Bacillus subtilis*	*Arabidopsis*	*Pseudomonas syringae*	Pure compound	[50]
Iturin A	*Bacillus subtilis*	*Arabidopsis jar1-1*	*Pseudomonas syringae*	Pure compound	[50]
Surfactin and fengycin	*Bacillus subtilis*	Bean	*Botrytis cinerea*	Mutant	[41]
Fengycin and iturin	*Bacillus velezensis*	Rice	*Pyricularia oryzae*	Mutants	[55]
Surfactin, fengycin, and iturin	*Bacillus velezensis*	Rice	*Pyricularia oryzae*	Mutants	[55]
Massetolide A	*Pseudomonas fluorescens*	Tomato	*Phytophthora infestans*	Mutant; pure compound	[56]
Sessilin	*Pseudomonas sessilinigenes*	Bean	*Rhizoctonia solani*	Mutants; pure compound	[57]
Orfamide	*Pseudomonas sessilinigenes*	Bean	*Rhizoctonia solani*	Mutants; pure compound	[57]
Orfamide	*Pseudomonas protegens*	Rice	*Cochliobolus miyabeanus*	Pure compound; mutant	[58]
WLIP	*Pseudomonas putida*	Rice	*Magnaporthe oryzae*	Mutants	[59]
Sessilin and orfamide	*Pseudomonas sessilinigenes*	Bean	*Rhizoctonia solani*	Mutants	[57]

## Data Availability

This work is theoretical/review-based; no new data were created or analyzed. Consequently, data sharing is not applicable.
